# Comparative Genomics-Based Evaluation of *Lacticaseibacillus rhamnosus* KFOM 0134 as a Probiotic against *Staphylococcus aureus*

**DOI:** 10.4014/jmb.2606.06012

**Published:** 2026-08-04

**Authors:** Yoon-Soo Gwak, Hui-yeon Suh, Hyeong-Rak Yoon, Somyeong Kim, Chan-Il Bae, Mi-Ju Kim

**Affiliations:** Institute of Life Sciences & Resources and Department of Food Science and Biotechnology, Kyung Hee University, Yongin 17104, Republic of Korea

**Keywords:** Probiotics, Anti-*Staphylococcus aureus*, Whole-genome sequencing, Comparative genomics, Antimicrobial peptides

## Abstract

Lactic acid bacteria are widely recognized for their probiotic potential and their ability to inhibit pathogenic microorganisms through the production of antimicrobial compounds and competitive exclusion mechanisms. *Staphylococcus aureus* is a globally significant human and animal foodborne pathogen and a major cause of foodborne diseases, posing serious public health concerns. This study aimed to develop a probiotic strain capable of suppressing *S. aureus*. *Lacticaseibacillus rhamnosus* strain KFOM 0134 was isolated from salted kimchi cabbage; it demonstrated strong tolerance to acidic and high-bile conditions. It did not exhibit any cytotoxic effects, but displayed significant anti-inflammatory activity and enhanced intestinal adhesion capacity. Notably, KFOM 0134 exerted pronounced antimicrobial activity against *S. aureus*, highlighting its potential as a functional probiotic. Whole-genome sequencing revealed bacteriocin- and cryptic antimicrobial peptide (AMP)-related genetic determinants, but not any virulence- or transferable antibiotic resistance-associated genes, supporting its pathogenic safety. To further contextualize this strain within species-wide diversity, 402 *L. rhamnosus* genomes were compared, revealing extensive diversity characterized by an open pangenome structure and clade-associated metabolic differentiation. Phylogenetic positioning and lineage-specific functional signatures provided additional insight into the genetic basis of antimicrobial competence. Overall, phenogenomics of *L. rhamnosus* KFOM 0134 confirmed its well-defined probiotic potential *via* experimental validation of functional traits, comprehensive genome-based safety assessments, and the identification of bacteriocin- and AMP-associated genes. These findings substantiate its safety and antimicrobial functionality, supporting its applicability in food safety management and functional food development.

## Introduction

With increasing health awareness and consumer recognition of the close relationship between diet and health, the scope of potential applications and practical uses of lactic acid bacteria (LAB) in health research has expanded, attracting considerable scientific interest [1, 2]. LABs are Gram-positive, nonspore-forming, anaerobes or facultative anaerobes that are naturally found in diverse environments; they play a key role in the fermentation of foods, including dairy products, meat, vegetables, and human gastrointestinal tract function [3]. LABs exert diverse probiotic functions, including modulation of gut microbiota, enhancement of intestinal barrier integrity, and enhancement of immunomodulatory effects, thereby contributing to host health [4]. Probiotic strains primarily belong to the genera *Lactobacillus*, *Bifidobacterium*, *Streptococcus*, *Leuconostoc*, *Pediococcus*, and *Enterococcus*, with safety evaluations indicating no major concerns regarding their use in foods and as dietary supplements [5].

In contrast, food contamination with pathogenic microorganisms presents a major global public health concern; the World Health Organization estimates that 600 million people fall ill, and 4.2 million die annually [6, 7]. The genus *Staphylococcus* comprises > 50 species that commonly colonize the skin and mucous membranes of humans and other animals; *Staphylococcus aureus* is one of the most important human and animal pathogens and a leading cause of foodborne diseases worldwide [8]. *S. aureus* is highly virulent and clinically significant whose extensive repertoire of toxins and virulence factors facilitates effective colonization, tissue invasion, and immune evasion, leading to infections that range from superficial skin and soft tissue infections to severe and life-threatening conditions such as pneumonia, endocarditis, osteomyelitis, septic arthritis, and bacteremia [9, 10]. Despite the development of various therapeutic and preventive strategies, *S. aureus* remains an important human pathogen because of its ability to colonize diverse host environments and cause a wide range of infections [11]. These characteristics emphasize the need for additional strategies to control the growth and pathogenic potential of *S. aureus*. In this context, probiotics and their antimicrobial metabolites, including antimicrobial peptides, have attracted attention as potential biological control agents. These bioactive compounds may inhibit pathogen growth and adhesion, interfere with biofilm formation, enhance intestinal barrier function, and modulate host immune responses, thereby providing a potential complementary approach for controlling *S. aureus*.

However, the antimicrobial efficacy of probiotics is highly strain-specific, reflecting inherent differences and underscoring the importance of systematic screening and mechanistic characterization [12]. Therefore, it is essential to delineate strain-specific properties comprehensively, given the inherent functional diversity observed among probiotics. In particular, genomics is required to identify and elucidate antimicrobial genetic determinants, including bacteriocin and AMP biosynthesis gene clusters, which play critical roles in probiotic-mediated inhibition of pathogenic bacteria [13]. Furthermore, comparative genomics of closely related strains provides valuable insights into the molecular basis of functional heterogeneity, enabling the identification of strain- or lineage-specific genes and pathways associated with antimicrobial activity [14, 15]. Such an integrative approach combining phenotypic screening, genome-wide analysis, and comparative genomics facilitates a mechanistic understanding of probiotic functionality, supporting the rational selection and development of probiotic strains with enhanced and predictable antimicrobial potential.

Therefore, this study isolated LABs from salted kimchi cabbage and systematically evaluated their probiotic potential. The isolates were further screened to identify strains exhibiting superior probiotic characteristics and antimicrobial activity against *S. aureus*. The selected probiotic strain was genetically analyzed employing whole-genome sequencing (WGS), with particular emphasis on safety assessment and identification of genetic determinants associated with anti-*S. aureus* activity. Furthermore, a comprehensive comparative analysis was conducted using publicly available genome sequences of 402 *Lacticaseibacillus rhamnosus* strains obtained from open-source databases. It elucidated strain- and clade-specific genetic characteristics, resolved phylogenetic relationships, and characterized clade-associated metabolic functional traits. Collectively, this integrative phenogenomics approach provides a robust framework for mechanistically linking antimicrobial activity to genomic features and supporting the rational selection and development of probiotic strains with defined and enhanced antimicrobial potential.

## Materials and Methods

### Primary Isolation of Microbial Strains and Selection of Probiotic Candidates

In this study, LABs were isolated from salted kimchi cabbage (Republic of Korea) by culturing samples : in de Man, Rogosa, and Sharpe (MRS) medium (Kisanbio Co., Ltd., Republic of Korea) for 48 h at 37°C under anaerobic conditions, and taxonomically identified through 16S rRNA gene sequencing. The isolated strains were preserved at -80°C in 25% (v/v) glycerol and reactivated by subculture before use.

Subsequently, candidate probiotic strains were selected based on their ability to tolerate bile salts and an acidic pH, criteria that are key for probiotic stability. The isolates were cultured overnight in MRS broth at 37°C and subcultured for a further 18 h. The cell concentration was then adjusted to 10^8^ CFU/mL using a UV-Vis spectrophotometer (GENESYS™ 180, Thermo Scientific, USA). Acid and bile tolerance were assayed according to the method described by Tabashiri *et al*. [2], with slight modifications. Bile tolerance was evaluated by inoculating each bacterial suspension at 1% (v/v) into MRS broth supplemented with 0.3% (w/v) Oxgall bile salts (Sigma-Aldrich, USA). The inoculated cultures were incubated at 37°C for 24 h under anaerobic conditions, and viable cell counts were determined before and after incubation by serial dilution and plating on MRS agar. For evaluating acid tolerance, MRS broth was similarly inoculated at 10% (v/v) microbial suspensions, and the pH values were adjusted to 2.5, 3.0, and 3.5 with HCl. Samples were collected at 0 h and at 3 h of incubation at 37°C. Subsequently, 1 mL aliquots were serially diluted, and 100 μL of each dilution was plated in triplicate on MRS agar. The plates were then incubated at 37°C for 48 h, and colonies were counted to determine the viable cell counts. The survival rate (%) was calculated as follows,

$$\text{Survival rate (\% )=}\frac{\text{log CFU/mL at 3h}}{\text{log CFU/mL at 0h}}\times 100$$

The strains exhibiting high tolerance to acidic pH and bile salts were further analyzed.

### Evaluation of Anti-*Staphylococcus aureus* Activity

**Agar well diffusion assay.** *S. aureus* ATCC 23235 served as a representative foodborne pathogenic bacterium for antimicrobial assays; it was cultured on tryptic soy agar (TSA) at 37°C for 24 h. Subsequently, the culture supernatant of *L. rhamnosus* KFOM 0134 was collected, neutralized with NaOH, and filtered through a 0.22 μm nylon membrane filter (Millipore) to obtain the cell-free supernatant (CFS). In parallel, cell pellets were washed twice with phosphate-buffered saline (PBS) and prepared as bacterial cell suspensions (BCSs). Overnight-cultured *S. aureus* ATCC 23235 was adjusted to 1 × 10^6^ CFU/mL and spread on TSA plates. Gentamicin (10 mg/mL) and MRS broth were used as positive and negative controls, respectively. CFSs and BCSs were plated and incubated at 37°C for 24 h. Antimicrobial activity was evaluated by measuring the diameter of the inhibition zones.

**Broth microdilution assay.** Antimicrobial activity was further evaluated using a broth growth inhibition assay. BCSs and CFSs were prepared following the procedure described previously. Sterile distilled water and CFSs were dispensed into a 96-well microplate; two-fold serial dilutions were employed to obtain a range of test concentrations, and MRS broth was used as a negative control. The pathogen culture was finally adjusted to 1 × 10^6^ CFU/mL, and 100 μL was added to each well. The plates were incubated at 37°C for 16 h; bacterial growth was monitored by measuring the optical density at 600 nm (OD_600_).

**Coculture assay.** The coculture assay followed a previously described method [7], with minor modifications. To prepare the coculture medium, equal volumes (5 mL each) of double-strength MRS broth and double-strength TSB were mixed to obtain MRS–TSB broth. *S. aureus* and KFOM 0134 were adjusted to 1 × 10^8^ CFU/mL and used to inoculate the MRS–TSB broth at 1% (v/v). Each strain was cultured individually in MRS or TSB under the same conditions as controls. The cultures were incubated at 37°C, and samples were collected at 0, 8, 16, and 24 h. Serial dilutions were plated on MRS agar and TSA to enumerate viable cells. The survival and growth rates of each organism during coculture and corresponding monoculture controls were compared.

### Evaluation of the Probiotic Properties of LAB

**Nitric oxide (NO) production assay.** To analyze its probiotic properties, KFOM 0134 was cultured overnight at 37°C, washed twice with PBS, and subsequently freeze-dried before further analyses. The murine macrophage cell line—RAW 264.7—was obtained from the Korean Cell Line Bank (KCLB, Republic of Korea) and cultured in Dulbecco’s Modified Eagle Medium (DMEM; Gibco™, USA) supplemented with 10% fetal bovine serum and 1% penicillin–streptomycin at 37°C in a 5% CO_2_ incubator. The anti-inflammatory effects of KFOM 0134 were evaluated in RAW 264.7 cells by measuring NO production. Cells cultured in complete DMEM medium were seeded at 5 × 10^5^ cells/well in 24-well plates, and NO production was stimulated with 100 ng/mL lipopolysaccharide (LPS). Subsequently, the CFS of KFOM 0134 was applied to the cells at concentrations ranging from 2.5 to 0.1 mg/mL, while 10 μM dexamethasone was used as a positive control. After incubation for 24 h, CFSs were collected; NO was quantified by mixing equal volumes of the supernatant and Griess’s reagent: 0.1% *N*-(1-naphthyl)ethylenediamine dihydrochloride, 1% sulfanilamide, and 2% H_3_PO_4_. The OD_540_ was measured with a microplate reader (Molecular Devices, USA). The results were normalized to the untreated negative control (UNTR) and are presented as NO concentrations (μM).

**MTT assay-based cell viability assessment.** The viability of RAW 264.7 macrophages was assessed employing the thiazolyl blue tetrazolium bromide (MTT) assay, indicating possible cytotoxic effects of KFOM 0134. Cells were seeded in culture plates at 5 × 10^5^ cells/well and allowed to adhere. Subsequently, the cells were incubated with the CFS at concentrations ranging from 2.5 to 0.1 mg/mL for 24 h at 37°C under a humidified atmosphere containing 5% CO_2_. Then, the cells were gently washed twice with PBS, MTT reagent was added to a final concentration of 0.1 mg/mL, and incubated for 4 h at 37°C to allow formazan crystal formation. They were subsequently solubilized in dimethyl sulfoxide, and the OD_570_ was measured with a microplate reader to determine cell viability.

**Assessment of intestinal adhesion using Caco-2 cells.** The human colon adenocarcinoma cell line Caco-2, obtained from KCLB, was used to evaluate the human intestinal epithelium adhesion capacity of LAB strains, based on the method described by Jin *et al*. [16] with minor modifications. The cells were seeded in 24-well plates and cultured at 37°C in a 5% CO_2_ incubator until 80%–90% confluence was reached. Subsequently, *L. rhamnosus* GG (ATCC 53103) and KFOM 0134 were added to the cells to a final concentration of 1 × 10^8^ CFU/mL, and incubated for 24 h. Next, each well was washed twice with PBS to remove nonadherent bacteria. Bacterial adhesion was quantified via crystal violet staining—the stain was solubilized in 33% acetic acid—and the OD_595_ was measured.

**Hemolytic activity assessment.** To assess hemolytic activity, TSA was prepared using 15.0 g of pancreatic digest of casein, 5.0 g of soy meal papaic digest, 5.0 g of NaCl, 15.0 g of agar, and 1 L of distilled water, supplemented with 5% (v/v) sheep blood to produce TSA blood agar. KFOM 0134 was streaked on TSA blood agar plates, incubated at 37°C for 48 h; ATCC 23235 was included as a positive control. Hemolytic activity was interpreted based on the criteria: α-hemolysis, characterized by a partial oxidation of hemoglobin to methemoglobin resulting in greenish coloration around colonies; β-hemolysis, indicated by the complete lysis of red blood cells producing a clear zone surrounding colonies; and γ-hemolysis, defined by the absence of any detectable hemolytic activity [17].

**Antibiotic resistance profiling.** To determine the antibiotic susceptibility of KFOM 0134, the minimum inhibitory concentration (MIC) was evaluated following the European Food Safety Authority (EFSA) guidelines [18]. Ampicillin, gentamicin, kanamycin, streptomycin, erythromycin, clindamycin, tetracycline, and chloramphenicol were used for the assay. KFOM 0134 was inoculated into 96-well plates at 5 × 10^4^ CFU/well, and antibiotics were added following twofold serial dilutions. The plates were then anaerobically incubated at 37°C for 48 h, and MICs were determined based on the EFSA-recommended MIC cut-off values.

### Genomic Characterization of Probiotic Strains

**Genomic DNA extraction and whole-genome sequencing.** For whole genome analysis of KFOM 0134, genomic DNA (gDNA) was extracted using the DNeasy Blood & Tissue Kit (Qiagen, Germany). The gDNA purity was assessed with a NanoReady Touch spectrophotometer (Hangzhou LifeReal Biotechnology Co., Ltd., China) and quantified utilizing the Qubit™ dsDNA HS Assay Kit on a Qubit™ 4 fluorometer (Invitrogen, USA). DNA samples with an OD_260/280_ ratio between 1.8 and 2.1 were subsequently analyzed. gDNA was repaired employing the NEBNext^®^ FFPE™ DNA Repair Mix (New England Biolabs [NEB], USA) and end prepared with the NEBNext^®^ Ultra II™ End Repair/dA-Tailing Module (NEB). Subsequently, DNA molecules were ligated with native barcodes, pooled, and ligated to sequencing adapters. The library was loaded onto a MinION Flow Cell R10.4.1 and sequenced on a Nanopore MinION Mk1B platform (Oxford Nanopore Technologies, UK). From the raw sequencing data, reads corresponding to ~100× coverage of the *L. rhamnosus* genome were selected for downstream analyses. They were basecalled using Dorado basecaller in a super-accuracy mode, barcode trimmed, and those with a minimum Q-score threshold of 20 were quality filtered utilizing NanoPlot v1.42.0 [19].

**Genome assembly and functional annotation.** The filtered reads were genome assembled *de novo* using Flye, and quality was evaluated using QUAST [20, 21]. Genes were annotated, and coding sequences (CDSs) were predicted utilizing Prokka and RAST; Clusters of Orthologous Groups (COG), Gene Ontology, and Kyoto Encyclopedia of Genes and Genomes (KEGG) pathways were functionally analyzed employing eggNOG-mapper [22-24]. BAGEL4 was used to mine ribosomally synthesized and posttranslationally modified peptides and bacteriocin gene clusters [25]. Subsequently, the resulting proteins were analyzed utilizing Macrel to identify cryptic candidate AMPs. The “AMPSphere” module was applied to detect AMP-like regions embedded within larger CDSs based on sequence similarity to known AMPs. Identified cryptic AMPs were mapped to their corresponding CDSs [26]. To evaluate the safety of the selected strain, antibiotic resistance genes were identified by accessing the Comprehensive Antibiotic Resistance Database (CARD), and potential virulence factors were recognized by referring to the Virulence Factors Database (VFDB) [27, 28].

### Comparative *L. rhamnosus* Genomic Analyses

**Genome-wide comparative analysis.** A total of 402 genomes, including that of KFOM 0134 and publicly available RefSeqs—obtained from the National Center for Biotechnology Information (NCBI)—were compiled to characterize the features of *L. rhamnosus* genomes *via* comparative genomics. Average nucleotide identity (ANI) was estimated using FastANI to evaluate genome-wide similarity among strains and to clarify their genetic relatedness [29]. Pan-genomics was conducted utilizing Roary to characterize common and strain-specific gene repertoires; the resulting profiles were visualized employing Anvi’o [30, 31]. Subsequently, phylogenetic tree construction, core genome alignment, and single-nucleotide polymorphisms (SNPs) were detected utilizing Parsnp v2.0 to elucidate evolutionary relationships among strains [32]

**Clade-specific genomic features.** Based on phylogenetic classification, the genomes were grouped into six distinct clades, and core genes within each were identified independently to define conserved gene sets. Such core genomes were functionally annotated using eggNOG-mapper to characterize KEGG modules and to elucidate conserved metabolic pathways and functional traits that characterize each clade. In parallel, AMPs were predicted utilizing Macrel to systematically assess their clade-associated repertoires, enabling comparative analyses of the presence, diversity, and distribution patterns of candidate AMPs across clades. Additionally, insights into potential clade-dependent antimicrobial capabilities were obtained.

### Statistical Analysis

All experiments were performed using three independent biological replicates, with each measurement conducted in technical triplicate unless otherwise stated. Data are presented as the mean ± standard deviation (SD). Statistical analyses were performed using SPSS version 28.0 (IBM, USA), and data were visualized employing GraphPad Prism version 9.0 (GraphPad Software, USA). For comparisons involving more than two groups, one-way analysis of variance (ANOVA) followed by Tukey’s honestly significant difference post hoc test was performed when the assumptions of normality and homogeneity of variance were satisfied, with adjusted *p*-values used for multiple comparisons, statistical significance defined as *p* < 0.05, and different lowercase letters above the bars indicating significant differences among groups. Furthermore, genomics and data presentation were accomplished using the R packages pheatmap, ggplot2, and ggtree to systematically illustrate genomic relationships, functional patterns, and phylogenetic structures across the datasets analyzed [33, 34].

## Results and Discussion

### Identification of Microbial Strains and Selection of Candidate Probiotics

**Fig. 1:**
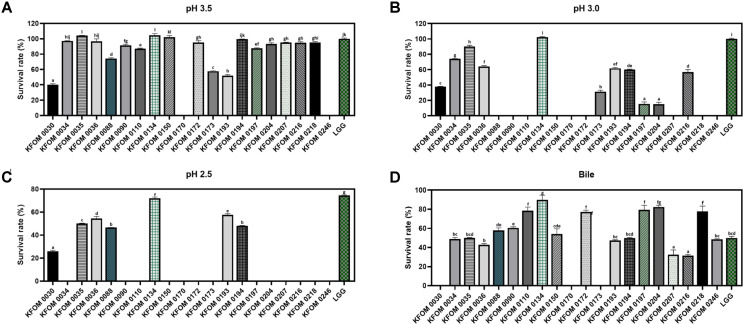
Acid and bile tolerance under simulated gastrointestinal conditions. Survival of the isolated strains was evaluated under acidic conditions at pH (**A**) 3.5, (**B**) 3.0, and (**C**) 2.5, as well as (**D**) in the presence of bile salts. Cell viability was expressed as the percentage of surviving cells relative to the initial viable count.

Salted kimchi cabbage, the primary raw material of kimchi, is produced by adding cabbage with 2% (w/v) NaCl; it harbors diverse microorganisms, including LAB [35]. This study isolated diverse species of LABs from kimchi and subsequently analyzed 20 representative strains (Table S1). Probiotics encounter various stresses during food processing and gastrointestinal transit. Their stress response mechanisms are species- and strain-dependent. Acid and bile tolerance are widely recognized essential selection criteria, as they reflect the ability of microorganisms to survive gastric acidity and small intestine bile salts, critical for their *in vivo* persistence and functional efficacy [36, 37]. Strains exhibiting superior tolerance to acidic conditions—pH 2.5, 3.0, and 3.5—and 0.3% (w/v) Oxgall bile salts were selected as candidate probiotics. As shown in Fig. 1, among all strains tested at pH 3.5 and 3.0, KFOM 0134 exhibited the highest survival rate, which was comparable to or significantly exceeded that of LGG (Fig. 1A and 1B). Under a more acidic condition at pH 2.5, both KFOM 0134 and LGG maintained the highest survival rates among the strains (Fig. 1C). In contrast, under a 0.3% bile salt condition, KFOM 0134 showed a markedly higher survival rate (89.7 ± 5.0%) than LGG, indicating superior bile tolerance (Fig. 1D). KFOM 0134 was selected for subsequent investigations and detailed functional analyses based on these capabilities.

### Evaluation of Anti-*Staphylococcus aureus* Activity

**Fig. 2:**
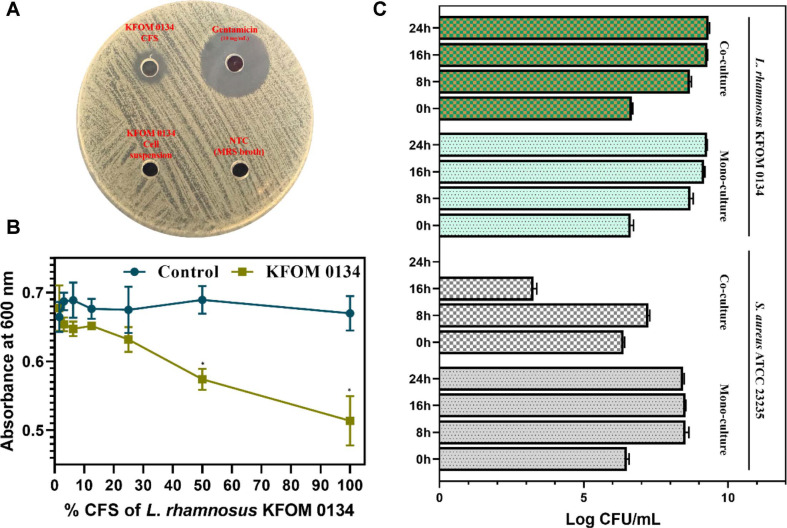
Activity of *L. rhamnosus* KFOM 0134 against *S. aureus*. (**A**) Agar well diffusion assay showing *S. aureus* inhibition zones produced by KFOM 0134. (**B**) Broth microdilution assay evaluating *S. aureus* growth inhibition by the CFS of KFOM 0134. (**C**) Co-culture assay demonstrating the *S. aureus* suppressive effects of KFOM 0134 during competitive growth.

The antibacterial activity of each strain was assessed against the major foodborne pathogen *S. aureus* ATCC 23235 via the agar well diffusion assay. The CFS of *L. rhamnosus* KFOM 0134 produced a clear inhibition zone—with a mean diameter of 12 mm—indicating notable antimicrobial capacity (Fig. 2A). Subsequently, the antimicrobial activity of KFOM 0134 against *S. aureus* was further evaluated utilizing the broth microdilution assay. To minimize any potential influence of organic acids, particularly lactic acid, the CFS pH was preadjusted to 6.5. As shown in Fig. 2B, CFS group significantly inhibited *S. aureus* more prominently than the control group, demonstrating marked bacterial growth suppression. Furthermore, such potential of KFOM 0134 was assessed *in vitro* through a coculture experiment. KFOM 0134 and *S. aureus* were inoculated at 6.54 ± 0.13 log(CFU/mL). *S. aureus* in monoculture grew normally, reaching 8.45 ± 0.02 log(CFU/mL) after 24 h. In contrast, during coculture with KFOM 0134, *S. aureus* proliferation initially increased to 7.28 ± 0.05 log (CFU/mL) at 8 h; however, it gradually declined thereafter, and no viable cells were detected after 24 h (Fig. 2C). Based on these findings, it can be assumed that KFOM 0134 demonstrated potent bactericidal activity against *S. aureus*. Together with its probiotic characteristics, KFOM 0134 has strong potential as a functional probiotic for controlling foodborne pathogens, modulating pathogen colonization, and enhancing host-associated microbial balance.

### Evaluation of the Probiotic Properties of KFOM 0134

**Fig. 3:**
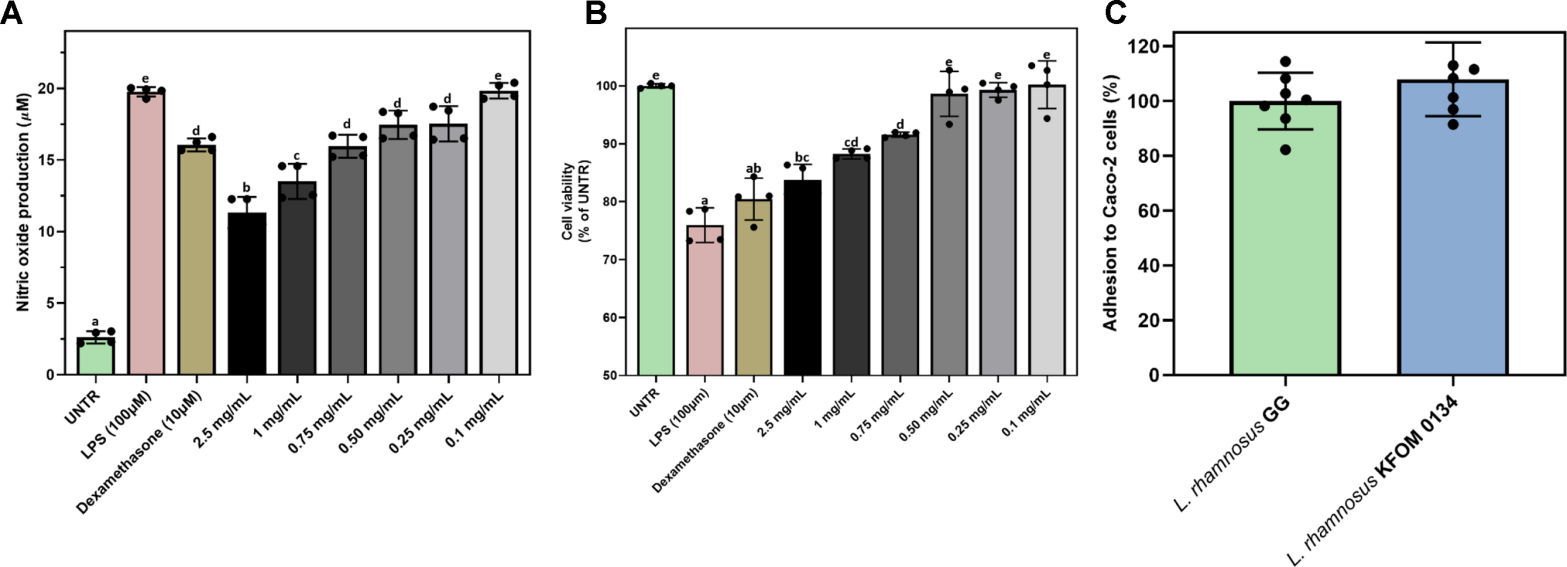
Functional characterization of the probiotic properties of *L. rhamnosus* KFOM 0134. (**A**) Suppression of NO production in LPS-stimulated macrophages, indicating anti-inflammatory activity. (**B**) Cell viability following treatment with KFOM 0134 was assessed by the MTT assay. (**C**) Adhesion capacity of KFOM 0134 to human intestinal epithelial Caco-2 cells.

Since NO is a key inflammatory mediator produced by activated macrophages, KFOM 0134 was further evaluated for its anti-inflammatory effects by measuring NO production in LPS-stimulated RAW 264.7 cells. LPS-stimulated cells exhibited a marked increase in NO production (19.77 μM) compared with the UNTR group (2.26 μM). In contrast, NO production was significantly reduced in dexamethasone-treated cells compared with the LPS-treated group (Fig. 3A). Compared with the LPS-treated group, treatment with the CFS of KFOM 0134 at concentrations ranging from 2.5 to 0.25 mg/mL significantly suppressed NO production to 11.32–17.53 μM, respectively. Notably, the maximum concentration lowered NO to levels less than those observed in the dexamethasone-treated group. However, no significant variation in NO production was observed at lower concentrations, compared with the LPS-treated control. Collectively, these results demonstrate that KFOM 0134 exhibits potent anti-inflammatory activity.

To determine whether these effects were attributable to cytotoxicity, the biosafety of the strain was further evaluated using MTT assays. LPS treatment reduced cell viability by 75.9 ± 0.2% compared with the UNTR (Fig. 3B). In contrast, KFOM 0134 at all concentrations tested maintained elevated cell viability > 80% relative to the UNTR samples. These findings indicate that the anti-inflammatory impacts observed were not due to cytotoxicity, confirming the strain’s safety under the test conditions.

In addition, to evaluate the intestinal adhesion capacity of the strain, Caco-2 cells were used. The adhesion capacity of KFOM 0134 was evaluated in comparison with the reference strain LGG (Fig. 3C). KFOM 0134 exhibited an adhesion rate 107.9 ± 13.4% greater compared to LGG, demonstrating higher levels of attachment to Caco-2 cells. These results indicate that KFOM 0134 possesses superior intestinal adhesion capability, suggesting enhanced intestinal colonization and persistence potential.

To assess the safety characteristics of KFOM 0134 for probiotic application, pathogenic potential and clinically relevant antibiotic resistance were ruled out by systematically examining hemolytic activity and antibiotic susceptibility. Hemolytic assays revealed that KFOM 0134 exhibited γ-hemolysis, indicating a nonhemolytic phenotype and supporting its contextual safety (Fig. S1). Antibiotic susceptibility analysis indicated that KFOM 0134 was resistant to gentamicin, kanamycin, and streptomycin (Table S2). WGS was subsequently conducted for further investigations to elucidate whether the resistance phenotype was intrinsic or associated with transferable resistance determinants.

### Genomic Features of KFOM 0134

**Fig. 4:**
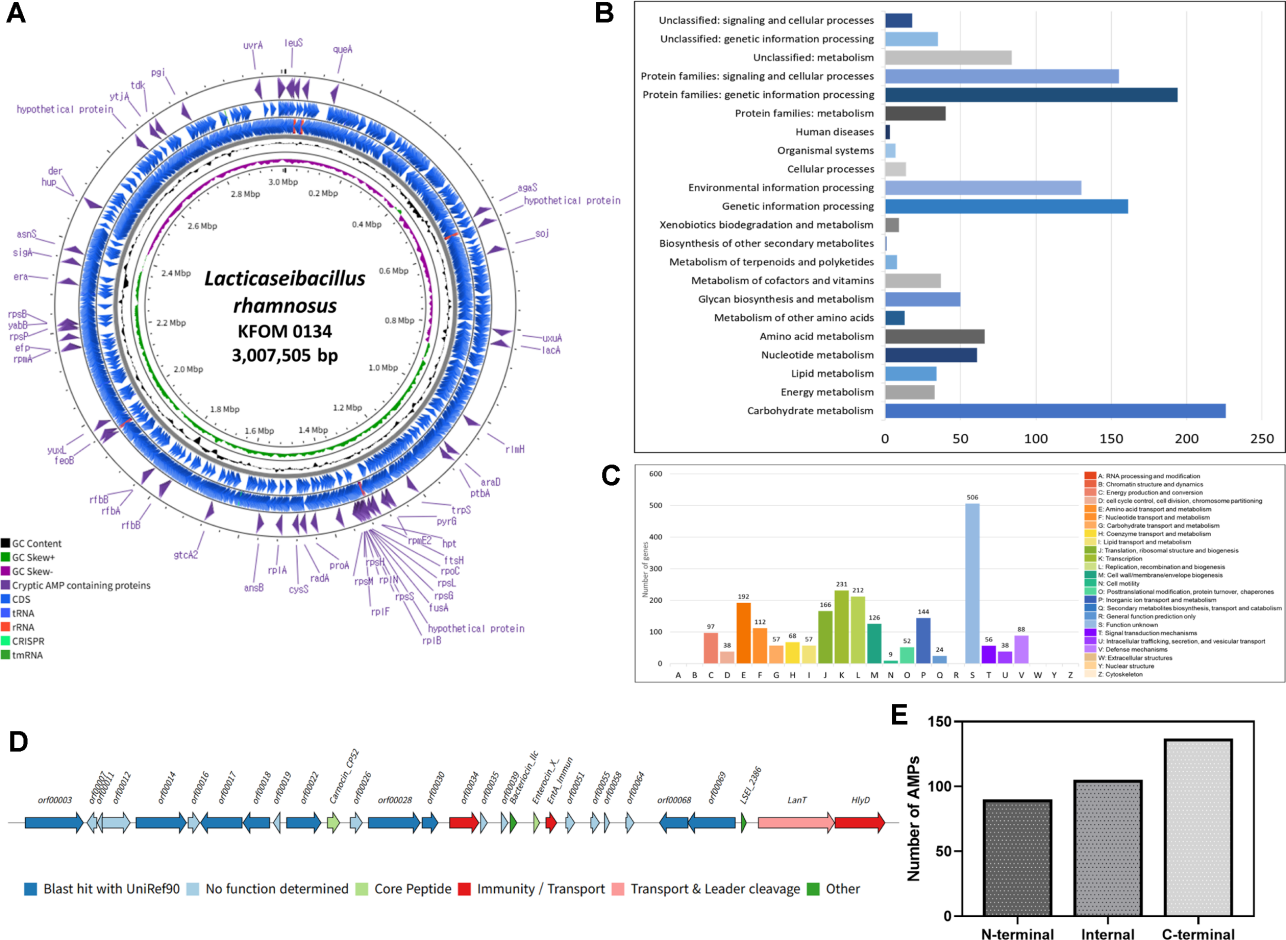
Genomic characteristics of *L. rhamnosus* KFOM 0134. (**A**) Circular map of the *L. rhamnosus* KFOM 0134 chromosome generated using the Proksee visualization tool. From the outer to the inner ring, the distribution of cryptic AMP-containing proteins, coding sequences (CDSs) on the forward and reverse strands, RNA genes, GC content, and GC skew are shown. (**B**) Functional classification of genes according to the KEGG database. (**C**) COG functional classification of the KFOM 0134 genome. (**D**) Bacteriocin-related genes were identified based on the whole-genome sequence of strain KFOM 0134 and utilizing the BAGEL4 program; they indicate its genetic potential for bacteriocin production, which may contribute to antimicrobial activity. (**E**) Positional distribution of cryptic AMPs within precursor proteins, categorized as N-terminal, internal, or C-terminal regions based on their relative starting positions along the precursor amino acid sequence.

Nanopore-based WGS was employed to genetically characterize *L. rhamnosus* KFOM 0134, and the resulting genome sequence has been deposited in the NCBI database under BioProject accession number PRJNA1494386 and BioSample accession number SAMN61601185. The KFOM 0134 genome was assembled into a single contig with a total length of 3,007,505 bp, indicating a complete and high-quality organization. Genome annotation identified 2,809 protein-coding, 15 rRNA, 59 tRNA, and 1 tmRNA gene (Fig. 4A). In addition, functional classification of genes employed the KEGG (Fig. 4B), revealing a high proportion of genes associated with carbohydrate metabolism, indicating the strain’s robust capacity for carbohydrate utilization and energy production. In addition, a substantial number of genes were annotated to the amino acid metabolism pathways, suggesting metabolic versatility and potential for active amino acid biosynthesis and transformation. As shown in Figure 4C, COG functional categorization revealed a high representation of genes associated with DNA transcription, replication, recombination, and repair, followed by category E: amino acid transport and metabolism. Such an enrichment suggests active gene regulation and genomic stability, as well as metabolic versatility [38]. In addition, those related to inorganic ion transport and metabolism were abundant. They may contribute to environmental adaptability and antimicrobial-based competition by maintaining metal ion homeostasis and modulating stress response mechanisms [39].

Genome mining using BAGEL4 was performed to investigate the presence of bacteriocin-associated gene clusters and evaluate the antimicrobial potential of the strain. Core peptides corresponding to Carnocin C52 and Enterocin X were identified (Fig. 4D). Carnocin Cp52 is a class IIa bacteriocin within Group III (subgroup III-1), whose members demonstrate bactericidal activity against several Gram-positive food-borne pathogens, including *Staphylococcus* spp. [40]. Notably, a bacteriocin IIc-associated determinant was also detected; it mediates the recognition of certain targets by class IIa bacteriocins and serves as a crucial species-specificity-governing factor [41]. Enterocin X is a class IIb (two-peptide) bacteriocin; IIb enterocins possess strong antimicrobial activity against major foodborne pathogens, including *S. aureus* [42]. Cryptic AMPs are derived from precursor proteins that may or may not be inherently antimicrobial, and such activity is often attributed to their cationic nature, which disrupts the microbial cell membranes [43]. Certain regions of precursor proteins, particularly the C-terminus, are positively charged [44]. Cryptic AMPs were predicted using Macrel to comprehensively investigate their antimicrobial potential at the whole-genome level. Based on thresholds of ≥ 80% sequence identity and ≥ 95% coverage, AMP mining identified 332 putative cryptic candidate AMPs encoded by 68 CDSs. When classified per a 30% positional threshold, the predicted AMP coding regions were distributed as follows- 90 (27.1%) in the N-terminal, 105 (31.6%) in the internal, and 137 (41.3%) in the C-terminal regions (Fig. 4A and 4E). These findings indicate that KFOM 0134 harbors multiple putative bacteriocin- and AMP-related genes, suggesting its genomic potential to produce antimicrobial compounds. Consistent with this potential, the strain demonstrated marked *in vitro* bactericidal activity against *S. aureus* ATCC 23235, suggesting that the predicted genes may contribute to the observed antimicrobial activity.

Subsequently, the KFOM 0134 genome was analyzed using CARD and VFDB to assess the presence of antimicrobial resistance and virulence-associated genes, respectively, applying thresholds of ≥80% sequence identity, ≥70% query coverage, and an E-value of ≤1 × 10^−5^; no genes meeting these predefined criteria were identified. Furthermore, despite phenotypic resistance to gentamicin, kanamycin, and streptomycin, corresponding acquired or transferable antibiotic resistance genes were not detected. These findings suggest that such a phenotype is likely intrinsic rather than associated with mobile genetic elements. Collectively, these genomic analyses revealed that KFOM 0134 possesses diverse functional and antimicrobial property-related genes while lacking virulence- and transferable antibiotic resistance- associated genes. These findings support the suitability of KFOM 0134 as a probiotic strain at a genetic-level.

### Genome-Wide Comparative Analysis

**Fig. 5:**
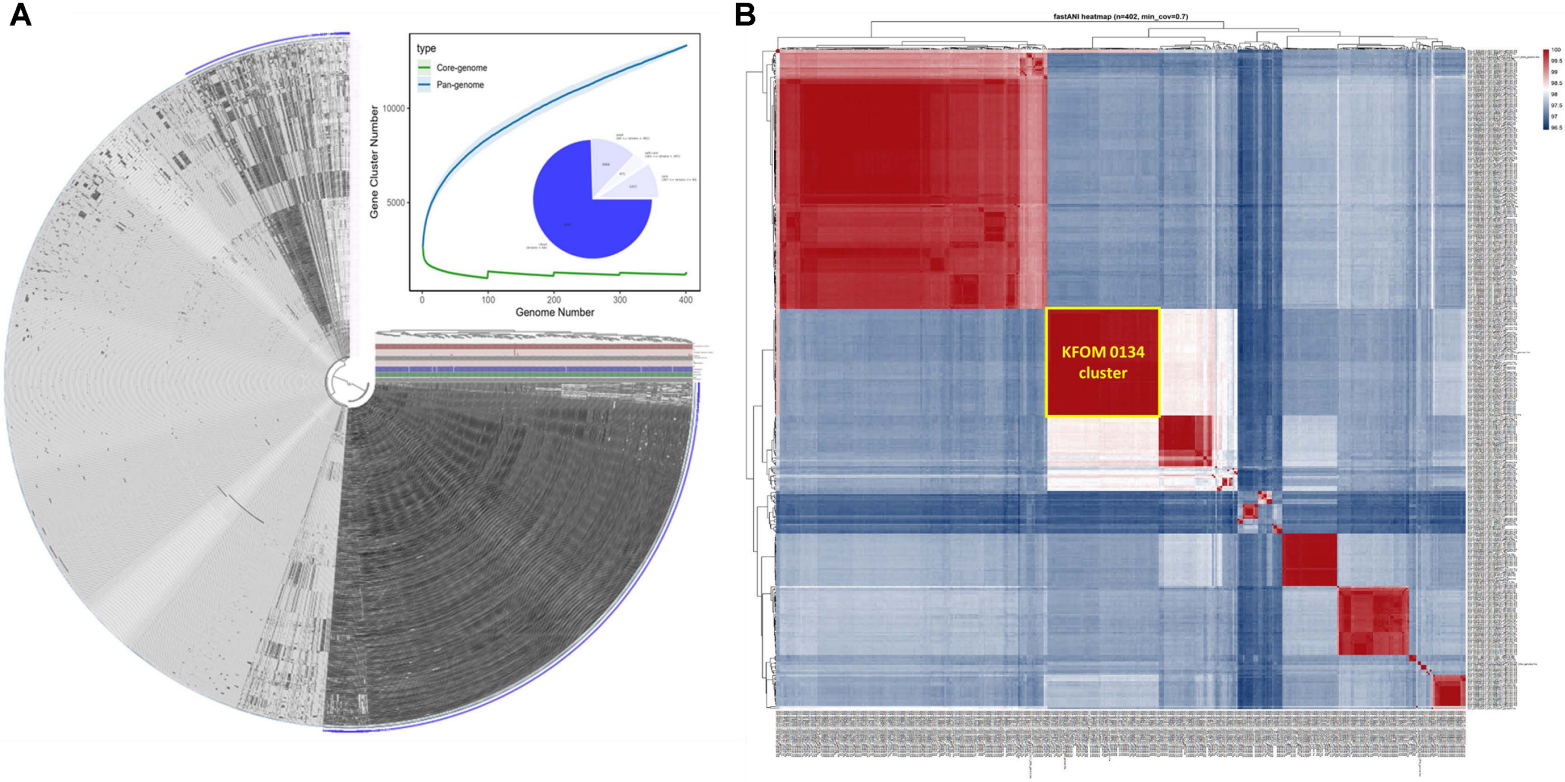
Pangenome structure and genomic relatedness among 402 *L. rhamnosus* strains. (**A**) Pangenome analysis was performed using Roary and visualized with Anvi’o; it showed the distribution of core and accessory genes as well as the pangenome accumulation curve across the strains analyzed. (**B**) ANI heatmap illustrating genomic similarity and clustering patterns among the strains.

To investigate the genomic characteristics of 402 *L. rhamnosus* strains, a comprehensive comparison was conducted. Pan-genomics was performed with Roary to evaluate the distribution of core, accessory, and strain-specific genes; the results were visualized using Anvi’o. In total, 13,339 distinct orthologs were recognized across 402 genomes. Among these, core genes present in 397–402 strains accounted for 9.4%, soft-core genes detected in 381–396 strains comprised 3.5%, shell genes identified in 60–380 strains represented 9.4%, and cloud genes recognized in ≤ 60 strains constituted 74.6% of the total pool (Fig. 5A). Anvi’o further illustrated extensive gene content variations across genomes, with a limited proportion of conserved core gene clusters but a broad distribution of accessory genes. The circular gene presence/absence matrix highlights the dominance of strain-specific and low-frequency genes, supporting the enhanced proportion of cloud genes observed in the dataset. Moreover, the pangenome accumulation curve showed a continuous increase in gene cluster number with the addition of novel genomes, without reaching a plateau. This pattern indicates that *L. rhamnosus* possesses an open pangenome, suggesting ongoing gene acquisition and substantial interspecific genomic diversity. ANI was analyzed to further evaluate genomic homology among the 402 strains. All strains exhibited ANI values above the 95% species threshold, confirming correct taxonomy. The results presented as a heatmap revealed distinct genomic clusters formed on the basis of similarity (Fig. 5B). Strains with higher ANI values were grouped, indicating close genetic relatedness. Notably, KFOM 0134 clustered with 64 strains, suggesting that it belongs to a well-defined lineage within the species and shares genomic features common to this subgroup.

**Fig. 6:**
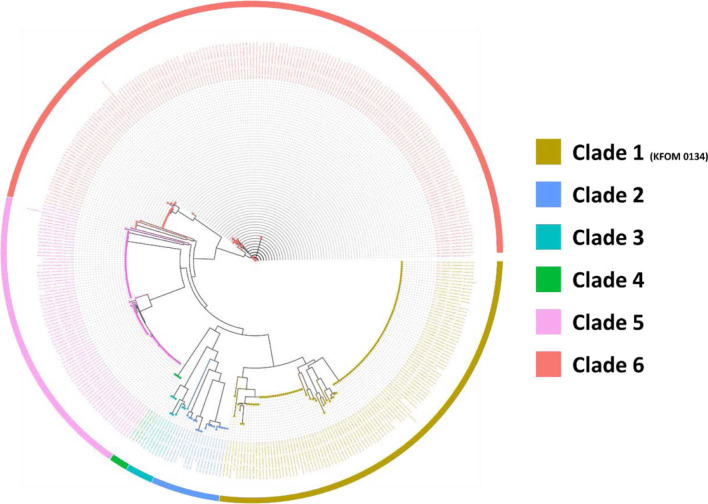
Core genome SNP-based phylogenetic analysis of 402 *L. rhamnosus* strains. A phylogenetic tree was constructed based on the SNPs identified within the core genome of each strain, revealing their evolutionary relationships. The strains were clustered into six distinct clades according to the SNP-based phylogeny, highlighting genomic divergence and lineage-specific grouping among the analyzed strains.

To investigate evolutionary relationships and genomic variations among strains, core genomes were aligned, and SNPs were detected *via* Parsnp v2.0. Based on the core SNPs identified, a phylogenetic tree was constructed to infer strain-level relationships (Fig. 6). To objectively delineate the population structure, cophenetic distances extracted from the Parsnp phylogeny were subjected to average-linkage hierarchical clustering, resolving the strains into six phylogenetically coherent clades.

### Clade-Specific Genomic Features

**Fig. 7:**
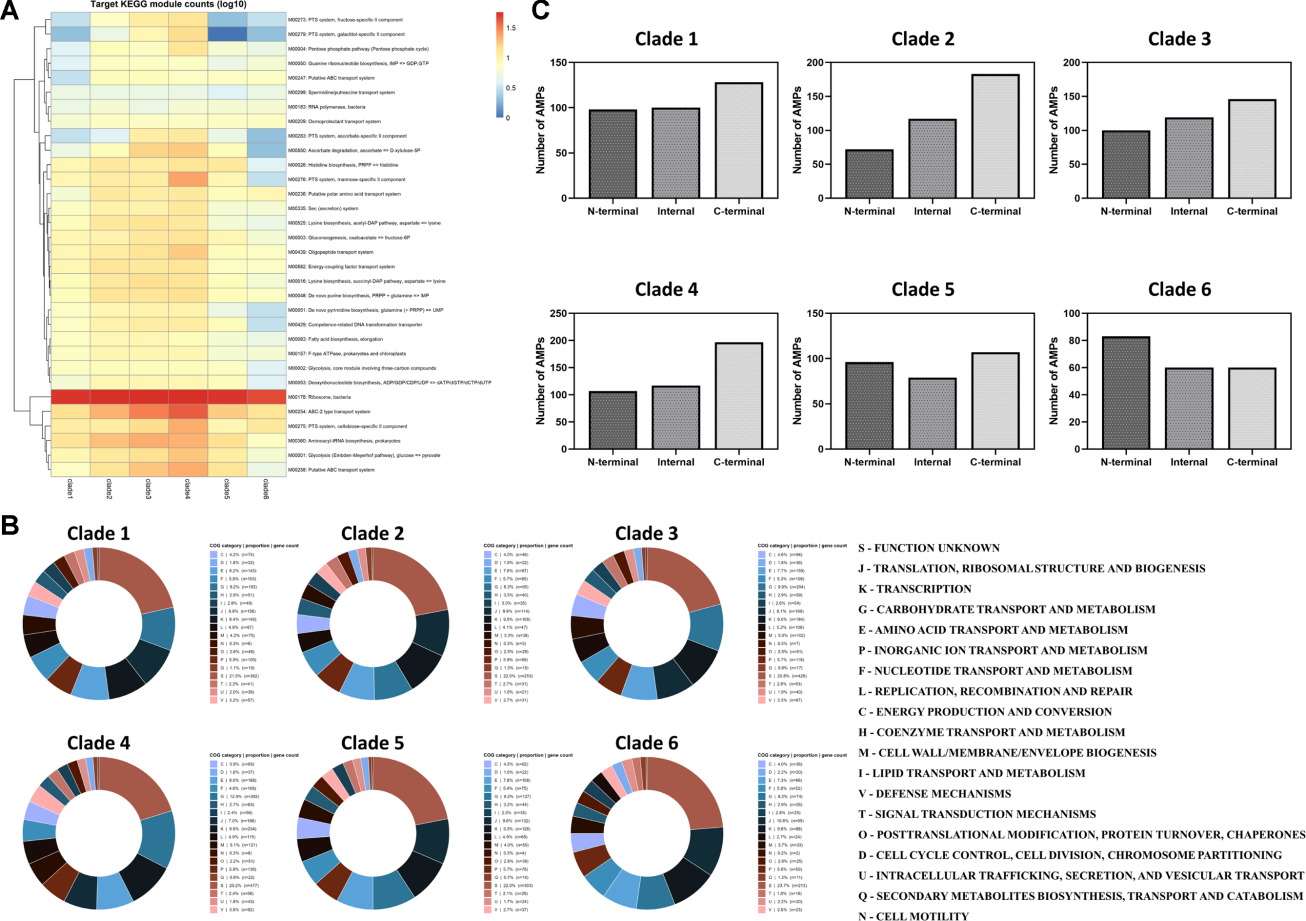
Clade-specific comparative genomics based on core genes. To investigate gene variations among the six clades, clade-level core genes were identified and compared. (**A**) Functional differentiation among clades based on a comparative analysis of KEGG modules. (**B**) COG profiling illustrating clade-specific genomic features and functional divergence. (**C**) Comparison of the positional distribution of cryptic AMPs within precursor proteins across the six clades.

Subsequently, the core genes from each clade were independently extracted and annotated utilizing KEGG module analysis to identify conserved clade-level metabolic pathways, lineage-associated functional traits, and genomic characteristics. KEGG module profiling revealed that while essential modules such as ribosome and central nucleotide biosynthesis were uniform across clades, transport systems and carbohydrate metabolism modules displayed clade-dependent variations (Fig. 7A). Clade 4 exhibited an elevated abundance of PTS system and ABC transporter modules, indicating greater metabolic diversification. KFOM 0134 was assigned to Clade 1, which had a comparatively more consistent distribution of metabolic modules without the pronounced expansion of specific functional categories, reflecting a functionally stable genomic framework. COG functional dissemination indicated that categories associated with information storage and processing—including translation (J), transcription (K), replication, recombination and repair (L), and RNA processing (A)—were highly conserved across clades, signifying a preservation of essential cell processes and genome maintenance systems (Fig. 7B). In contrast, divergence was primarily observed in metabolism- and transport-related categories, indicating clade-dependent functional distinction. Notably, Clade 1, which included KFOM 0134, displayed a comparatively invariable and proportionally balanced functional composition without any remarkable enrichment of specific metabolic pathways. This finding suggests that Clade 1 retained a stable and evolutionarily conserved genomic constitution, reflecting functional robustness rather than lineage-specific metabolic expansion or niche-driven specialization. To assess whether cryptic AMP distribution is associated with phylogenetic lineage, the relative abundance of candidate AMPs across clades was analyzed according to their location within precursor proteins (Fig. 7C). Overall, the candidates were predominantly prevalent in C-terminal regions across most clades. This trend was consistent in Clade 1, suggesting that C-terminal bias represents a lineage-level genomic feature rather than a strain-specific phenomenon. In contrast, Clade 6 displayed a unique AMP dissemination pattern characterized by a greater proportion of N-terminal-derived cryptic AMPs, deviating from the general species-wide trend. These findings indicate that although C-terminal enrichment appears to be a conserved structural tendency within *L. rhamnosus*, clade-specific variations in AMP abundance and positional bias are also evident. Such preferential localization may be linked to an enrichment of positively charged residues within the C-terminal domain, which could enhance antimicrobial potential. Collectively, comparative genomics revealed substantial diversity within *L. rhamnosus*, characterized by an open pangenome structure and obvious phylogenetic clustering. Clade-level differences in metabolic pathways and cryptic AMP repertoires highlight lineage-associated functional specialization. These findings provide a genomic framework for understanding strain-level variations and support the functional relevance of KFOM 0134 within its lineage.

## Conclusion

This study isolated *L. rhamnosus* KFOM 0134 from salted kimchi and systematically evaluated its probiotic potential through integrated phenotypic and genomic analyses. The strain demonstrated strong tolerance to acidic and bile-rich conditions, significant anti-inflammatory activity without any detectable cytotoxicity, and pronounced antimicrobial effects against *S. aureus*, indicating its functional robustness under physiologically relevant conditions. WGS confirmed the absence of virulence and transferable antibiotic-resistance-associated genes while simultaneously identifying bacteriocin- and cryptic AMP-related determinants. These genomic features provide a molecular basis for the safety and antimicrobial functionality of the strain. Furthermore, comparative genomics involving 402 *L. rhamnosus* strains revealed extensive diversity characterized by an open pangenome structure and clade-level functional differentiation. Clade-specific metabolic signatures and lineage-associated AMP distributions underscore evolutionary interspecific diversification and contextualize the genome-based phylogenetic position of KFOM 0134. Collectively, these findings indicate that KFOM 0134 possesses desirable probiotic characteristics, *in vitro* antimicrobial activity against *S. aureus* ATCC 23235, and a favorable genomic safety profile. This comprehensive evaluation supports its suitability as a promising probiotic candidate for applications in food safety, functional foods, and pathogen control strategies.

## Supplemental Materials

Supplementary data for this paper are available on-line only at http://jmb.or.kr.
