# Supplementary material for: Comparative Genomics-Based Evaluation of Lacticaseibacillus rhamnosus KFOM 0134 as a Probiotic against Staphylococcus aureus
Source: J Microbiol Biotechnol. 2026 Aug 4;36:e2606012. doi: 10.4014/jmb.2606.06012 (PMC13486750; doi:10.4014/jmb.2606.06012)
Supplement: Supplementary file 1 [file jmb-36-e2606012-supple.pdf]

J. Microbiol. Biotechnol. 2026. 36: e2606012

https://doi.org/10.4014/jmb.2606.06012

Supplementary Tables and Figure

Table S1. Taxonomic identification and characteristics of the 20 representative lactic acid bacterial strains used in this study.

Number	Strain	Species
1	KFOM 0030	*Levilactobacillus koreensis*
2	KFOM 0034	*Weissella cibaria*
3	KFOM 0035	*Leuconostoc citreum*
4	KFOM 0036	*Latilactobacillus sakei*
5	KFOM 0088	*Leuconostoc Kimchii*
6	KFOM 0090	*Latilactobacillus sakei*
7	KFOM 0110	*Latilactobacillus sakei* subsp. *sakei*
8	KFOM 00134	*Lacticaseibacillus rhamnosus*
9	KFOM 0150	*Leuconostoc citreum / Leuconostoc holzapfelii*
10	KFOM 0170	*Leuconostoc mesenteroides* subsp. *dextranicum*
11	KFOM 0172	*Leuconostoc citreum*
12	KFOM 0173	*Lactobacillus curvatus*
13	KFOM 0193	*Leuconostoc citreum*
14	KFOM 0194	*Leuconostoc mesenteroides* subsp. *jonggakjibkimchii*
15	KFOM 0197	*Leuconostoc mesenteroides* subsp. *mesenteroides*
16	KFOM 0204	*Leuconostoc mesenteroides* subsp. *Jonggajibkimchii*
17	KFOM 0207	*Latilactobacillus sakei*
18	KFOM 0216	*Leuconostoc citreum*
19	KFOM 0218	*Latilactobacillus sakei* subsp. *sakei*
20	KFOM 0246	*Weissella koreensis*

Table S2. Antibiotic susceptibility profile of *L. rhamnosus* KFOM 0134 determined by minimum inhibitory concentration (MIC) according to EFSA guidelines.

Antibiotic	MIC (mg/L)	Cut-off value (mg/L) [a]	Susceptibility [b]
Ampicillin	<0.5	4	S
Gentamicin	64	16	R
Kanamycin	128	64	R
Streptomycin	64	32	R
Erythromycin	1	1	S
Clindamycin	1	1	S
Tetracycline	<2	8	S
Chloramphenicol	4	4	S

[a] Cut-off value was based on European Food Safety Authority (EFSA) recommendations.

[b] S = susceptible; R = resistance

(A) *S. aureus* ATCC 23235 (B) *L. rhamnosus* KFOM 0134

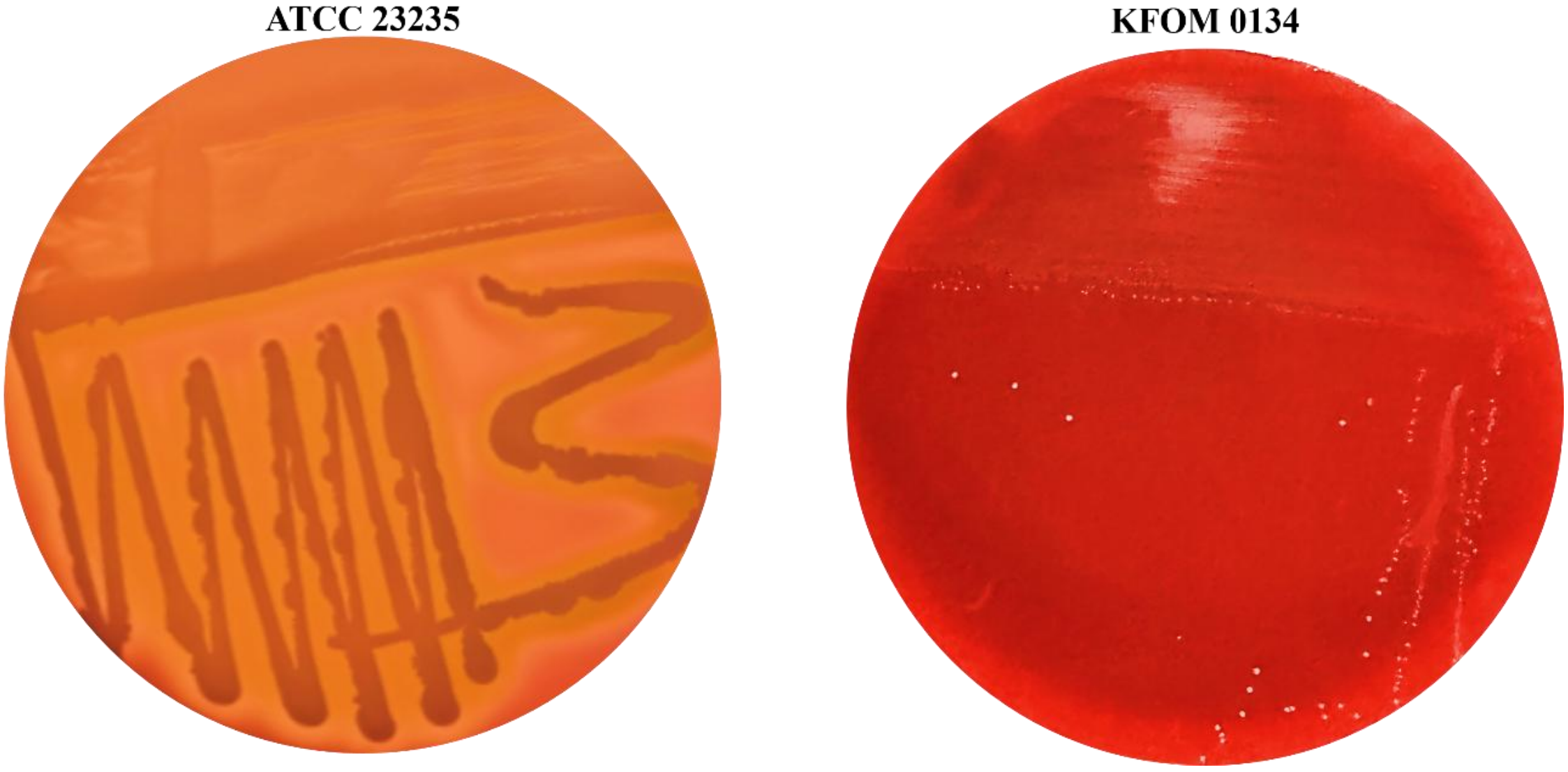

Fig. S1. Hemolytic activity of (A) *S. aureus* ATCC 23235, (B) *L. rhamnosus* KFOM 0134.

Hemolysis pattern observed on 5% sheep blood agar after incubation at 37°C for 48 h.
